# Complete plastid genome sequence of *Daucus carota*: Implications for biotechnology and phylogeny of angiosperms

**DOI:** 10.1186/1471-2164-7-222

**Published:** 2006-08-31

**Authors:** Tracey Ruhlman, Seung-Bum Lee, Robert K Jansen, Jessica B Hostetler, Luke J Tallon, Christopher D Town, Henry Daniell

**Affiliations:** 1 Dept. of Molecular Biology & Microbiology, University of Central Florida, Biomolecular Science, Building #20, Room 336, Orlando, FL 32816-2364, USA; 2 Section of Integrative Biology and Institute of Cellular and Molecular Biology, Patterson Laboratories 141, University of Texas, Austin, TX 78712, USA; 3 The Institute for Genomic Research, 9712 Medical Center Drive, Rockville, MD 20850, USA

## Abstract

**Background:**

Carrot (*Daucus carota*) is a major food crop in the US and worldwide. Its capacity for storage and its lifecycle as a biennial make it an attractive species for the introduction of foreign genes, especially for oral delivery of vaccines and other therapeutic proteins. Until recently efforts to express recombinant proteins in carrot have had limited success in terms of protein accumulation in the edible tap roots. Plastid genetic engineering offers the potential to overcome this limitation, as demonstrated by the accumulation of BADH in chromoplasts of carrot taproots to confer exceedingly high levels of salt resistance. The complete plastid genome of carrot provides essential information required for genetic engineering. Additionally, the sequence data add to the rapidly growing database of plastid genomes for assessing phylogenetic relationships among angiosperms.

**Results:**

The complete carrot plastid genome is 155,911 bp in length, with 115 unique genes and 21 duplicated genes within the IR. There are four ribosomal RNAs, 30 distinct tRNA genes and 18 intron-containing genes. Repeat analysis reveals 12 direct and 2 inverted repeats ≥ 30 bp with a sequence identity ≥ 90%. Phylogenetic analysis of nucleotide sequences for 61 protein-coding genes using both maximum parsimony (MP) and maximum likelihood (ML) were performed for 29 angiosperms. Phylogenies from both methods provide strong support for the monophyly of several major angiosperm clades, including monocots, eudicots, rosids, asterids, eurosids II, euasterids I, and euasterids II.

**Conclusion:**

The carrot plastid genome contains a number of dispersed direct and inverted repeats scattered throughout coding and non-coding regions. This is the first sequenced plastid genome of the family Apiaceae and only the second published genome sequence of the species-rich euasterid II clade. Both MP and ML trees provide very strong support (100% bootstrap) for the sister relationship of *Daucus *with *Panax *in the euasterid II clade. These results provide the best taxon sampling of complete chloroplast genomes and the strongest support yet for the sister relationship of Caryophyllales to the asterids. The availability of the complete plastid genome sequence should facilitate improved transformation efficiency and foreign gene expression in carrot through utilization of endogenous flanking sequences and regulatory elements.

## Background

The plastid is a nearly autonomous organelle because it contains the biochemical machinery necessary to replicate and transcribe its own genome and carry out protein synthesis. Within angiosperms the plastid genome includes approximately 120 to 130 genes and usually ranges in size from 120 to 170 kilobases (kb) [1-3]. Of the estimated 3000 or so distinct proteins found in the higher plant plastid [4,5] only a small fraction are encoded by the plastid genome [6]. The bulk of the plastid proteome is nuclear encoded, translated on cytosolic ribosomes and subsequently translocated across the plastid envelopes [7].

The circular plastid genome is divided into four regions: large single copy (LSC), small single copy (SSC) and the inverted repeat (IR) which is present in exact duplicate separated by the two single copy regions. Restriction site analysis indicates that the molecule exists in two orientations present in equimolar proportions within a single plant [8]. The circular molecule undergoes interconversion into a dumbbell-shaped conformation that is facilitated by the IR. Concerted evolution within the IR [9,10] suggests intramolecular recombination between the repeats may be occurring.

The advantages of plastid transformation for bioengineering are several-fold and include the integration of multiple genes in a single transformation event [11-13], lack of gene silencing [14-16], position effect due to site-specific transgene integration [17], and minimization of pleiotropic effects due to compartmentalization of recombinant proteins [15,18,19]. The presence of many copies of the plastid genome within the many plastids in each cell contributes to high levels of foreign protein expression [14]. Plastid genetic engineering could minimize transgene escape because of maternal inheritance of transgenes [17,20-24] and the possibility of employing cytoplasmic male sterility to contain transgenes [25].

The ability to transform the plastid genome of higher plants has facilitated the accumulation of foreign proteins previously found to be recalcitrant in plant expression systems [26]. Until very recently breakthroughs in this regard have been limited to *Nicotiana tabacum *(tobacco) where plastid transformation has become routine [27,28]. As the field progresses to encompass the expression of vaccine antigens and other therapeutic proteins via the plastid genome [29], there is a growing interest in developing a crop system for oral delivery of these recombinant products. *Daucus carota *(carrot) has been proposed as an ideal candidate for this application for several reasons. Cultivated carrot is a biennial, the reproductive structures are not present until the second year, yet the root crop is suitable for harvest in the first year [30]. This feature further ensures the ability to contain foreign genes in the field by eliminating the possibility of dispersal by pollen and seed. In terms of storage, the root may be realistically maintained up to six months without any processing under typical commercial conditions [31]. With an average annual value of over 70 million dollars, carrot ranks in the top ten among commercial vegetable crops in the United States adding to the interest in biotechnological improvement of this species [31].

Transformation of the carrot plastid genome has been accomplished, and expression of betaine aldehyde dehydrogenase (BADH) from spinach in carrot plastids was found to confer salt tolerance up to 400 mM NaCl [32]. In this case native carrot plastid sequences flanking the integration site were amplified by PCR from primers derived from the tobacco genome, due to the scarcity of carrot plastid DNA sequences in the public databases. Despite the potential of plastid genetic engineering, this technology has only recently been extended to a few major crops, including soybean [33], carrot [32] and cotton [34], via somatic embryogenesis, achieving transgene expression initially via non-green plastids [28]. Most previous studies focused on direct organogenesis by bombardment of leaves containing mature green plastids [28].

Although overall gene content and order are highly conserved among land plants, this same conservation is not observed in non-coding sequences such as introns and intergenic spacers (IGS), which along with the untranslated regions of genes (UTRs), comprise about 50 % of the plastome [35-38]. Genes for input traits such as insect [14,39] and herbicide resistance [23], salt [32] and drought tolerance [15] and pathogen resistance [40] as well as output traits such as the production of therapeutic proteins [19,41-43] are targeted to IGS regions to avoid disruption of endogenous genes. Integration of foreign sequences is dependent on homologous recombination between the transformation vector and the plastid genome. It is possible to achieve integration without 100% sequence identity between the vector and plastid genome sequence but recombination and hence transformation efficiency is impaired when sequences are divergent [37,44]. Additionally, evaluations of UTRs from a variety of species indicate the need to employ species-specific regulatory elements, such as promoters and translation sequences, to elevate the level of foreign protein expression [45,46].

Completely sequenced plastid genomes also provide a valuable source of phylogenetic data for resolving relationships among angiosperms [35,47-50]. The use of DNA sequences from shared plastid genes provides many more characters for phylogeny reconstruction relative to previous molecular phylogenies based on one to several plastid genes. However, the use of complete plastid genome sequences is constrained because of limited taxon sampling, a phenomenon that can often lead to incorrect tree topologies [*e.g.*, [35,49,51-54]]. Thus, there is an increased need to expand taxon sampling of complete plastid genomes to overcome this problem. Currently there are 35 published plastid genome sequences of angiosperms [37,55]. Some major lineages have multiple genome sequences available, especially basal angiosperms, monocots, and rosids, whereas other major clades are represented by only one or two taxa. The euasterid II clade represents one lineage that is undersampled. This group, comprising four major subclades with approximately 35 families and 32,000 species [56], has only one published genome sequence from *Panax *[57,58].

In this paper, we report on complete plastid genome sequence of *Daucus carota*, the first sequenced member of the family Apiaceae. We describe the organization of this genome and we present a phylogenetic analysis of *Daucus *and 29 other angiosperm plastid genomes based on 61 shared protein-coding genes. This is only the second published plastid genome sequence of the species-rich euasterid II clade. The complete plastid genome sequence of *Daucus *also provides valuable information for the application of plastid genetic engineering to this economically important crop plant [46].

## Results

### Size, gene content, order and organization of the carrot plastid genome

**Figure 1:**
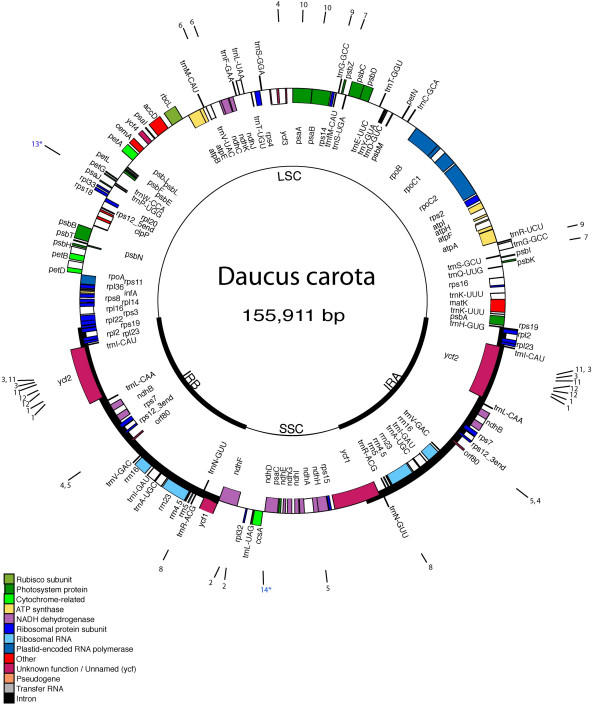
Map of the *Daucus carota *plastid genome. The thick lines indicate the extent of the inverted repeats (IRa and IRb), which separate the genome into small (SSC) and large (LSC) single copy regions. Genes on the outside of the map are transcribed in the clockwise direction and genes on the inside of the map are transcribed in the counterclockwise direction. Numbered ticks around the map indicate the location of repeated sequences found in the carrot genome; black = direct, blue = palindrome; * indicates that repeated sequence begins at the same position (see Table 2 for details).

The complete *Daucus carota *plastid genome is 155,911 base pairs (bp) in length (Fig. 1). The inverted repeat is 27,051 bp and the two copies are separated by two single copy regions; the large single copy region is 84,242 bp long and the small single copy region is 17,567 bp. There are a total of 136 predicted coding regions, 115 of which are unique and 21 are duplicated in the IR. On LSC/IRb boundary, the IR extends into *rps19*, resulting in the duplication of a portion of this gene. There are 81 unique protein-coding genes, 10 of which are duplicated in the IR. Also in the IR region is the ribosomal operon, which includes all 4 rRNA genes as well as tRNA-Ile and tRNA-Ala. There are five additional tRNAs within the IR resulting in a total of 37 tRNA genes, 30 of which are unique. There are 18 genes containing introns, with 15 of these with only a single intron (Table 1). Non-coding sequences, including IGS regions and introns, comprise 43.61 % of the carrot plastome. The overall nucleotide composition is 62.34 % AT and 37.66 %.

### Repeat analysis

Repeat analysis identified 12 direct repeats and 2 palindromes of ≥ 30 bp with a sequence identity of ≥ 90 % (Hamming distance of 3). Repeated sequences were found in IGS regions, introns and within coding sequence (Table 2). There are 4 direct repeats in *ycf2*, with repeated sequences ranging up to 70 bp in length.

### Phylogenetic analysis

Our phylogenetic data set included 61 protein-coding genes for 31 taxa (Table 3), including 29 angiosperms and two gymnosperm outgroups (*Pinus *and *Ginkgo*). The data set comprised 45,582 nucleotide positions but when the gaps were excluded to avoid regions with ambiguous alignment due to length variation there were 39,490 characters.

**Figure 2:**
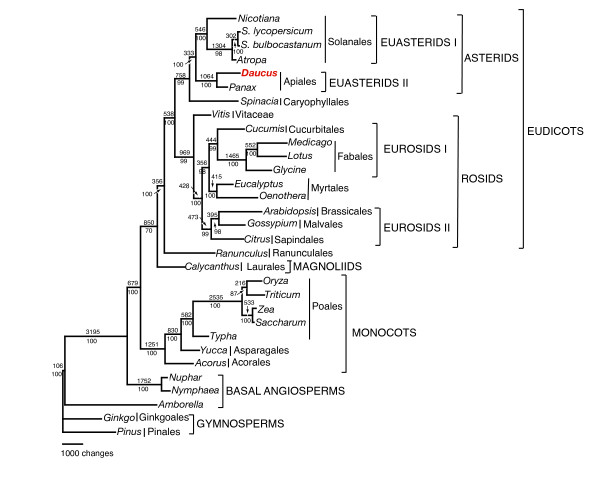
Phylogenetic tree of 31-taxon data set based on 61 plastid protein-coding genes using maximum parsimony. The tree has a length of 54,140, a consistency index of 0.44 (excluding uninformative characters) and a retention index of 0.60. Numbers above node indicate number of changes along each branch and numbers below nodes are bootstrap support values. Ordinal and higher level group names follow APG II [104]. Taxon in red is *Daucus*, the new genome reported in this paper.

**Figure 3:**
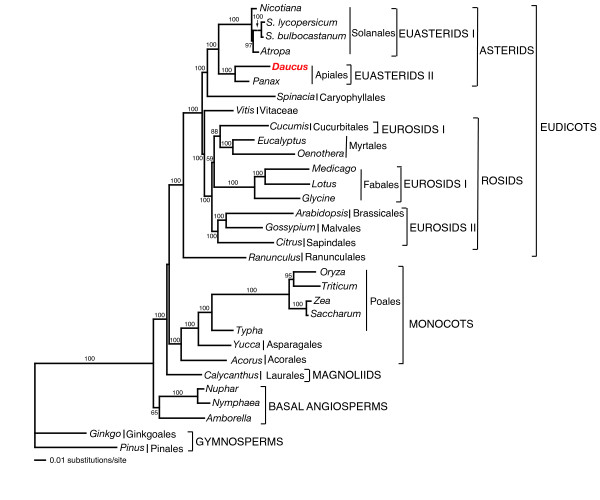
Phylogenetic tree of 31-taxon data set based on 61 plastid protein-coding genes using maximum likelihood. The tree has a ML value of – lnL = 312205.340. Numbers at nodes are bootstrap support values ≥ 50%. Ordinal and higher level group names follow APG II [104]. Taxon in red is *Daucus*, the new genome reported in this paper.

Maximum Parsimony (MP) analyses resulted in a single, fully resolved tree with a length of 54,140, a consistency index of 0.44 (excluding uninformative characters) and a retention index of 0.60 (Fig. 2). Bootstrap analyses indicated that 26 of the 28 nodes were supported by values ≥ 95% and 19 of these had a bootstrap value of 100%. Maximum likelihood (ML) analysis resulted in a single tree with – lnL = 312205.340. ML bootstrap values also were also high, with values of = 95% for 24 of the 28 nodes and 22 nodes with 100% bootstrap support. The ML and MP trees had very similar topologies but differed in three places. (1) The MP tree placed *Amborella *as the sister group to all other angiosperms, whereas the ML tree placed *Amborella *sister to the Nymphaeales, and together this group formed the sister group of all other angiosperms. Support for the relationships of basal angiosperms in the MP tree is strong (100%) but only moderate in the ML tree (65%). (2) The MP tree placed *Calycanthus *sister to the eudicots, whereas the ML tree positioned *Calycanthus *as sister to a large clade that included both monocots and eudicots. Support for the different placements of *Calycanthus *was weak in both MP and ML analyses. (3) Relationships among the rosids differed, especially the position of *Cucumis *and the monophyly of eurosids I. The MP tree (Fig. 2) provides strong support for the monophyly of the eurosid I clade because *Cucumis *is sister to the three legume taxa. In contrast, the ML tree (Fig. 3) places *Cucumis *sister to the two examined taxa of Myrtales, and support for this relationship is not as strong (88% bootstrap value). These three differences were detected in recent phylogenies based on complete plastid genome sequences of basal angiosperms [49] and rosids [35]. The remaining angiosperms formed two major clades, one including monocots and a second including the eudicots (Figs. 2, 3). Monophyly of the monocots was strongly supported (100% bootstrap value for both MP and ML). Ranunculales were strongly supported as sister to the remaining eudicots. There were two major clades of core eudicots, one including the rosids and the second including the Caryophyllales + asterids. Both MP and ML trees provide very strong support (100% bootstrap) for the sister relationship of *Daucus *with *Panax *in the euasterid II clade.

## Discussion

### Implications for plastid genetic engineering

An important agricultural crop worldwide, carrot has long attracted attention from the research community. It was the first crop species in which somatic embryogenesis was demonstrated [59]. This ability, to regenerate entire plants from cell or tissue cultures, has helped to maintain interest in carrot as our technology has advanced to include the improvement of agronomic species via genetic manipulation. The carrot nucleus has been the recipient of foreign genes to confer input characteristics such as pathogen resistance [60] and herbicide resistance [61]. Recently an extensive analysis of four *Agrobacterium rhizogenes *strains and twelve *Daucus carota *genotypes examined the utility of green fluorescent protein (GFP) as a selectable marker for nuclear transformants [62] demonstrating continued interest in this system for expression of foreign proteins.

Most interesting has been the exploration of carrot as an ideal platform for the production of proteins of significance to pharmacology. The small isoform of human glutamic acid decarboxylase (GAD65) has been identified as a major autoantigen contributing to the onset of insulin-dependent diabetes mellitus (IDDM) [63]. Expression of the GAD65 cDNA in transgenic carrot and tobacco resulted in an immunoreactive product which retained appropriate enzymatic function. Unfortunately levels of expression were quite low for both tobacco leaves and carrot taproots, on the order of 0.040 % and 0.012 % of total soluble protein (TSP), respectively [64]. A heterologous version of human GAD65 having the N-terminus substituted with GAD67 from rat was expressed in tobacco and was able to achieve stable accumulation of functional immunoreactive product up to 0.19% of the TSP. Although oral administration of disease associated autoantigens such as GAD65 can lead to the induction of tolerance in the murine model, dosage on the order of milligrams per week per mouse are required [65]. Expression levels and stable accumulation will have to be improved by orders of magnitude to make oral dosage truly feasible.

Vaccine antigens have been expressed in a number of plant species [66-69], including carrot. Transformation experiments have introduced a hemagglutinin glycoprotein [70] and a novel chimeric polyepitope antigen [71], both for neutralizing immunization against measles virus into the carrot nuclear genome. Extracted proteins demonstrated immunogenicity, raising antibody titers in sera of injected mice. However, quantitative data on protein accumulation in transgenic plants is lacking, especially for taproots. An estimate based on ELISA of 2% of membrane fraction in crude membrane preparations from carrot leaf is offered, but no mention is made of protein content in the root [70]. It is noteworthy that these extracts were homogenized with Freund's adjuvant (1:1) prior to initial injection and at each boost. Freund's adjuvant is employed to enhance antibody formation suggesting that plant extracts from nuclear transformants were insufficient to induce the desired immune response.

The need for an alternative expression system to obtain high levels of protein accumulation in carrot roots becomes apparent if efficacious oral delivery is to be accomplished. Furthermore a system for oral delivery of antigens should ideally include the adjuvant, limiting further the need for post harvest processing. Transformation of the plant plastid has demonstrated the capacity to produce substantial quantities of functional and immunoreactive proteins [reviewed in [29]].

Stable transformation of the *Daucus carota *plastid genome via somatic embryogenesis has been demonstrated recently [32]. Integration of foreign genes in these experiments was accomplished through the use of flanking sequences that were PCR-amplified from the native carrot plastid ribosomal operon, whereas the regulatory sequences used to facilitate expression of the transgenes were derived from tobacco and bacteriophage T7. When assayed for BADH enzyme function, roots of carrot plastid transformants showed activity up to 74.8 % of leaf tissue. In root tissues, plastome copy number is generally about 5 % of the level in mature leaves. The notably high activity is probably due to the elevated concentration of root chromoplasts in carrot, the plastid type responsible for the orange coloration. With the availability of the entire carrot plastid genome sequence, it will now be possible to incorporate native translation regulatory sequences into transformation constructs to further enhance foreign protein accumulation in carrot plastids. Additionally, detailed knowledge of this genome will allow the identification of optimal intergenic spacer regions for the integration of transgenes.

Receptor-mediated translocation of antigens and other pharmaceutical proteins across the intestinal mucosa offers the potential to make plant-produced, orally delivered vaccines and therapeutics a reality. The toxin of *Vibrio cholerae *(CT) is recognized as one of the most potent mucosal adjuvants. The holotoxin is composed of the A subunit, responsible for toxicity, and the non-toxic homopentameric B subunit (CTB), which facilitates entry into epithelial cells of the intestine by binding the GM1 receptor followed by endocytosis. Recombinant forms excluding the A subunit are rendered non-toxic, and when fused to another antigen, the B subunit cannot only carry this antigen across the intestine, but also strongly potentiate the antigen's immunogenicity [72,73]. Recently a fusion construct of CTB and GFP expressed in transplastomic tobacco demonstrated the efficacy of CTB to deliver foreign proteins to the circulatory system of mice, which were fed pulverized leaf tissue from plastid transformants. Between the two protein sequences investigators included the cleavage site for the ubiquitous protease furin to facilitate the intracellular cleavage of GFP. Quantitative ELISA revealed accumulation of CTB-GFP in transgenic plants ranging from 19.09 to 21.3% of TSP. Following oral administration of CTB-GFP expressing leaf material to mice, fluorescence microscopy and immunohistochemical analyses confirmed the presence of GFP in the mouse intestinal mucosa, liver and spleen while CTB remained in the intestinal cells [74]. Remarkable levels of protein accumulation coupled to a receptor-mediated oral delivery mechanism offers realistic hope for the possibility of plant-derived, orally delivered therapeutic proteins.

### Genome organization and evolution

The *Daucus *genome with two copies of an IR separating the SSC and LSC regions is identical in architecture to most sequenced angiosperm plastid genomes [2]. The size of the genome at 155,911 bp is also within the known range for angiosperms, which generally vary from 150,519 [75] to 162,686 bp [47] for taxa that have both copies of the IR. The size of the *Daucus *IR at 27,051 bp is at the upper end of the size range of other sequenced genomes, which vary from 23,302 (*Calycanthus*) [76] to 27,807 (*Oenothera*) bp [77]. Gene content and order of the *Daucus *plastid genome are identical to *Panax *[58], the only other published euasterid II genome.

A number of recent comparisons of plastid genomes of angiosperms have identified dispersed direct and inverted repeats [35-37,78]. The carrot genome contains similar numbers and sizes of repeats to these other angiosperms (Table 2, Fig. 1). In most cases, these repeats are located in intergenic spacer regions and in introns but several also occur in tRNAs and protein-coding genes. Examination of repeats in highly rearranged algal and angiosperm genomes have demonstrated a correlation between both the number and location of the repeats and the propensity for rearrangements [79,80]. The role of dispersed repeats in unrearranged plastid genomes remains unknown.

### Phylogenetic implications

The phylogenies based on 61 protein-coding plastid genes for 29 angiosperms (Figs. 2, 3) are largely congruent with relationships suggested by previous studies based single and multiple genes [56] and a number of recent phylogenies based on complete plastid genome sequences [35,36,48-50,76,81]. There is strong support for the monophyly the major clades of angiosperms, including monocots, eudicots, rosids, asterids, eurosids II, asterids I and asterid II. The three areas of incongruence between the MP and ML trees regarding relationships of basal angiosperms, *Calycanthus*, and eurosids I were identified previously [35,49], and are likely due to limited taxon sampling and misspecification of model parameters in large concatenated, multigene data sets.

The position of Caryophyllales within angiosperms has been controversial in the past. Previous molecular phylogenies clearly indicated that this order is a member of the eudicot clade [56], but relationships of Caryophyllales to other major eudicot lineages remains uncertain. The order has been suggested to be allied to rosids, asterids, or simply as an unresolved major eudicot clade sister to the Dilleniaceae [82]. Two recent phylogenies based on 61 shared plastid gene sequences provided support for a sister relationship between the Caryophyllales and asterids [35,36], however, only a single representative of the euasterid II clade was included. The addition of the *Daucus *plastid genome to this data set increases the level of support for a sister relationship of asterids and Caryophyllales (Figs. 2, 3).

Finally, the multiple plastid gene phylogenies also provide strong support for the monophyly of the euasterid II clade (Figs. 2, 3). This result is not surprising given that the Araliaceae (*Panax*) and Apiaceae (*Daucus*) have been considered sister families for a long time based on both morphological and molecular data [56]. Expanded taxon sampling of the other three major clades of euasterids II is needed to further test the monophyly and relationships of this large, diverse angiosperm clade.

## Conclusion

This is the first sequenced plastid genome of the family Apiaceae and only the second published genome sequence of the species-rich euasterid II clade. Both MP and ML trees provide very strong support (100% bootstrap) for the sister relationship of *Daucus *with *Panax *in the euasterid II clade. These results provide the best taxon sampling of complete chloroplast genomes and the strongest support yet for the sister relationship of Caryophyllales to the asterids.

The availability of the complete plastid genome sequence should facilitate improved transformation efficiency and foreign gene expression in carrot through utilization of endogenous flanking sequences and regulatory elements. The ability to express high levels of foreign protein, particularly those of clinical interest, makes plant plastids an attractive target for biotechnology. As a biennial crop which is amenable to relatively long term storage, carrot taproots may provide a feasible platform for the production of pharmaceutical proteins for oral delivery. The availability of the complete plastid genome sequence should facilitate improved transformation efficiency and foreign gene expression in carrot through utilization of endogenous flanking sequences and regulatory elements.

## Methods

### DNA isolation and amplification

*Daucus carota *L. cv half long plants were purchased fresh from a local market with leaves intact. Leaf tissue (10 g) was collected for plastid isolation based on the sucrose step gradient centrifugation method of Palmer [83]. Isolation was followed by whole plastid genome Rolling Circle Amplification (RCA) using the Repli-g RCA kit (Qiagen, Inc.) following the methods outlined in [84]. After incubation at 30°C for 16 hr, the reaction was terminated with 10-minute incubation at 65°C. Digestion of the RCA product with *BstXI, EcoRI *and *HindIII *allowed verification of successful RCA amplification of the plastome, as well as assessment of its quality prior to genome sequencing.

### DNA sequencing and genome assembly

DNA was sheared by nebulization, size fractionated to 4–6 kb, linker ligated and cloned into pHOS2, a TIGR medium copy vector. A total of 1231 high quality reads with an average length of 808 bases was generated during the random (1126 reads) and closure (105 reads) phases of sequencing. Sequences were assembled using TIGR assembler [85] and scaffolded using Bambus [86]. Sequence finishing included directed PCR to span gaps and directed primer walking of clones to cover the entire genome and to complete regions of low depth of coverage.

### Annotation and analysis of repeat structure

The *Daucus carota *plastid genome was annotated using DOGMA [87], which performs BLASTX searches against a custom database of previously published plastid genomes to identify putative coding sequences. The user submits a FASTA-formatted input file of the complete plastid genome sequence for analysis. DOGMA identified putative start and stop codons, which must then be confirmed by the user for each putative protein-coding gene. Identification of intron and exon boundaries and tRNAs and rRNAs must also be confirmed. The fully annotated plastid genome of *Daucus carota *was submitted to NCBI GenBank with the following accession number [GenBank:DQ898156 ].

Analysis of repeat structure was carried out using Comparative Repeat Analysis (CRA) [88]. The settings for identifying direct and inverted (palindromic) repeats included a size range between 30–5000 bp and a Hamming distance of 3 (limiting hits to sequence identity of ≥ 90%).

### Phylogenetic analysis

The 61 genes included in the analyses of Goremykin *et al. *[47], Leebens-Mack *et al. *[49], Lee *et al. *[36], and Jansen *et al. *[35] were extracted from the plastid genome sequence of *Daucus *using the DOGMA [87]. The same set of 61 genes was extracted from plastid genome sequences of 30 other sequenced plastid genomes (see Table 3 for complete list of genomes examined). All 61 protein-coding genes of the 31 taxa were translated into amino acid sequences, aligned using MUSCLE [89] followed by manual adjustments, and then nucleotide sequences of these genes were aligned by constraining them to the aligned amino acid sequences. A Nexus file with character sets for phylogenetic analyses was generated after nucleotide sequence alignment was completed. The complete nucleotide alignment is available online at Chloroplast Genome Database [90].

Phylogenetic analyses using maximum parsimony (MP) and maximum likelihood (ML) were performed with PAUP* version 4.10b10 [91]. Phylogenetic analyses excluded gap regions to avoid alignment ambiguities in regions with variation in sequence lengths. All MP searches included 100 random addition replicates and TBR branch swapping with the Multrees option. Modeltest 3.7 [92] was used to determine the most appropriate model of DNA sequence evolution for the combined 61-gene dataset. Hierarchical likelihood ratio tests and the Akaike information criterion were used to assess which of the 56 models best fit the data, which was determined to be GTR + I + Γ by both criteria. For ML analyses we performed an initial parsimony search with 100 random addition sequence replicates and TBR branch swapping, which resulted in a single tree. Model parameters were optimized onto the parsimony tree. We fixed these parameters and performed a ML analysis with three random addition sequence replicates and TBR branch swapping. The resulting ML tree was used to re-optimize model parameters, which then were fixed for another ML search with three random addition sequence replicates and TBR branch swapping. This successive approximation procedure was repeated until the same tree topology and model parameters were recovered in multiple, consecutive iterations. This tree was accepted as the final ML tree (Fig. 3). Successive approximation has been shown to perform as well as full-optimization analyses for a number of empirical and simulated datasets [93]. Non-parametric bootstrap analyses [94] were performed for MP analyses with 1000 replicates with TBR branch swapping, 1 random addition replicate, and the Multrees option and for ML analyses with 100 replicates with NNI branch swapping, 1 random addition replicate, and the Multrees option.

## Abbreviations

cpDNA, plastid DNA; IR inverted repeat; SSC, small single copy; LSC, large single copy, bp, base pair; plastome, plastid genome: ycf, hypothetical chloroplast reading frame; rrn, ribosomal RNA; MP, maximum parsimony; ML, maximum likelihood.

## Authors' contributions

SBL isolated plastids, performed RCA amplification of cpDNA, genome annotation, analysis and submission of data to the GenBank; TR performed the repeat analyses, drew the genome map and wrote some sections of the first draft; JBH, LJT and CDT performed DNA sequencing and genome assembly; RKJ assisted with extracting and aligning DNA sequences, performed phylogenetic analyses, and wrote the phylogenetic portions of the manuscript; HD conceived and designed this study, interpreted data, wrote and revised several versions of this manuscript. All authors read and approved the final manuscript.

## Figures and Tables

**Table 1:** Intron-containing genes found in the carrot plastome

**Protein coding**	**Transfer RNA**
*rps16*	*trn*-Lys (UUU)
*atpF*	*trn*-Gly (GCC)
*rpoC1*	*trn*-Leu (UAA)
*yf3*+	*trn*-Val (UAC)
*clpP*+	*trn*-Ala (UGC)*
*petB*	*trn*-Ile (GAU)*
*petD*	
*rpl16*	
rpl2*	
ndhB*	
rps12*+	
*ndhA*	

* genes are located in the IR; + genes contain two introns

**Table 2:** Repeats identified in the carrot plastid genome

Repeat #	Length	Location 1	Location 2
Direct			
1*	70	*ycf2 *(IRa,b)	*ycf2 *(IRa,b)
2	42	*ndhF*:*rpl32*	*ndhF*:*rpl32*
3*	34	*ycf2 *(IRa,b)	*ycf2 *(IRa,b)
4*	42	*ycf3 *intron 2	*rps12*_3end:*orf80 *(IRa,b)
5*	41	*rps12*_3end:*orf80 *(IRa,b)	*ndhA *intron
6	34	*ndhC*:*trnV*-UAC	*ndhC*:*trnV*-UAC
7	33	*trnS*-GCU	*trnS*-UGA
8*	31	*rrn5*:*trnR*-ACG (IRa,b)	*trnR*-ACG:*rrn5 *(IRa,b)
9	30	*trnG*-GCC	*trnG*-GCC
10	30	*psaB*	*psaA*
11*	30	*ycf2 *(IRa,b)	*ycf2 *(IRa,b)
12*	30	*ycf2 *(IRa,b)	*ycf2 *(IRa,b)
Palindrome			
13	30	*psaJ*:*rpl33*	*psaJ*:*rpl33*
14	30	*ccsA*:*ndhD*	*ccsA*:*ndhD*

Repeats at least 30 bp in size, with a sequence identity greater or equal than 90% are included.

Repeats marked with * are present in the IR and are therefore duplicated.

See Fig. 1 for location of repeats on the gene map.

**Table 3:** Taxa included in phylogenetic analyses with GenBank accession numbers and references

Taxon	GenBank Accession Numbers	Reference
Gymnosperm Outgroups		
*Pinus thunbergii*	NC_001631	Wakasugi *et al. *1994 [95]
*Ginkgo biloba*	DQ069337–DQ069702	Leebens-Mack *et al. *2005 [49]
Basal Angiosperms		
*Amborella trichopoda*	NC_005086	Goremykin *et al. *2003 [47]
*Nuphar advena*	DQ069337–DQ069702	Leebens-Mack *et al. *2005 [49]
*Nymphaea alba*	NC_006050	Goremykin *et al. *2004 [48]
Magnoliids		
*Calycanthus floridus*	NC_004993	Goremykin *et al. *2003 [76]
Monocots		
*Acorus americanus*	DQ069337–DQ069702	Leebens-Mack *et al. *2005 [49]
*Oryza sativa*	NC_001320	Hiratsuka *et al. *1989 [96]
*Saccharum officinarum*	NC_006084	Asano *et al. *2004 [97]
*Triticum aestivum*	NC_002762	Ikeo and Ogihara, unpublished
*Typha latifolia*	DQ069337–DQ069702	Leebens-Mack *et al. *2005 [49]
*Yucca schidigera*	DQ069337–DQ069702	Leebens-Mack *et al. *2005 [49]
*Zea mays*	NC_001666	Maier *et al. *1995 [98]
Eudicots		
*Arabidopsis thaliana*	NC_000932	Sato *et al. *1999 [99]
*Atropa belladonna*	NC_004561	Schmitz-Linneweber *et al. *2002 [100]
*Citrus sinensis*	DQ864733	Bausher *et al. *unpublished
*Cucumis sativus*	NC_007144	Plader *et al. *unpublished
*Daucus carota*	DQ898156	Current study
*Eucalyptus globulus*	AY780259	Steane 2005 [101]
*Glycine max*	NC_007942	Saski *et al. *2005 [78]
*Gossypium hirsutum*	NC_007944	Lee *et al. *[36]
*Lotus corniculatus*	NC_002694	Kato *et al. *2000 [75]
*Medicago truncatula*	NC_003119	Lin *et al.*, unpublished
*Nicotiana tabacum*	NC_001879	Shinozaki *et al. *1986 [102]
*Oenothera elata*	NC_002693	Hupfer *et al. *2000 [77]
*Panax schinseng*	NC_006290	Kim and Lee 2004 [58]
*Ranunculus macranthus*	DQ069337–DQ069702	Leebens-Mack *et al. *2005 [49]
*Solanum lycopersicum*	DQ347959	Daniell *et al. *[37]
*Solanum bulbocastanum*	NC_007943	Daniell *et al. *[37]
*Spinacia oleracea*	NC_002202	Schmitz-Linneweber *et al. *2001 [103]
*Vitis vinifera*	NC_007957	Jansen *et al. *2006 [35]
